# Advances in the joint profiling technologies of 5mC and 5hmC

**DOI:** 10.1039/d4cb00034j

**Published:** 2024-04-05

**Authors:** Bo He, Haojun Yao, Chengqi Yi

**Affiliations:** a Peking-Tsinghua Center for Life Sciences, Academy for Advanced Interdisciplinary Studies, Peking University Beijing China chengqi.yi@pku.edu.cn; b Peking University Chengdu Academy for Advanced Interdisciplinary Biotechnologies Chengdu China; c College of Chemistry and Chemical Engineering, Hunan University Changsha China; d State Key Laboratory of Protein and Plant Gene Research, School of Life Sciences, Peking University Beijing China; e Department of Chemical Biology and Synthetic and Functional Biomolecules Center, College of Chemistry and Molecular Engineering, Peking University Beijing China

## Abstract

DNA cytosine methylation, a crucial epigenetic modification, involves the dynamic interplay of 5-methylcytosine (5mC) and its oxidized form, 5-hydroxymethylcytosine (5hmC), generated by ten-eleven translocation (TET) DNA dioxygenases. This process is central to regulating gene expression, influencing critical biological processes such as development, disease progression, and aging. Recognizing the distinct functions of 5mC and 5hmC, researchers often employ restriction enzyme-based or chemical treatment methods for their simultaneous measurement from the same genomic sample. This enables a detailed understanding of the relationship between these modifications and their collective impact on cellular function. This review focuses on summarizing the technologies for detecting 5mC and 5hmC together but also discusses the limitations and potential future directions in this evolving field.

## Abstract 2

Overview of the joint profiling technologies of 5mC and 5hmC.

## Graphical abstract

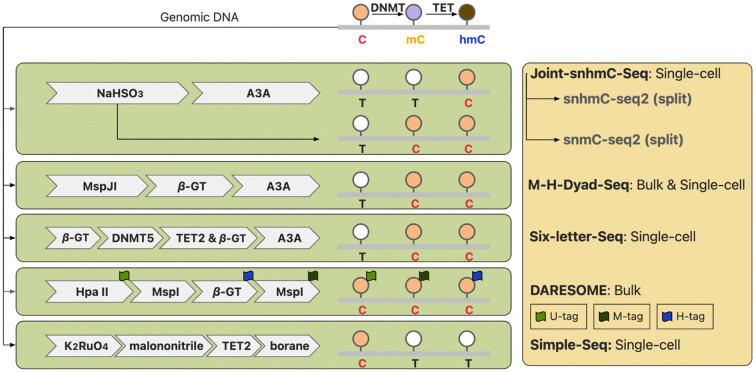

## 1. Introduction

5-Methylcytosine (5mC), often termed the fifth base, plays crucial biological roles like regulating tissue-specific gene expression and silencing retroviral elements. The formation of 5mC occurs when a methyl group is added to the 5th carbon of cytosine. This process is facilitated by DNA methyltransferases (DNMTs) and predominantly takes place within CpG dinucleotides.^1,2^ The ten-eleven translocation (TET) enzymes are known for their ability to iteratively oxidize DNA, leading to demethylation.^3–5^ This process results in the production of 5-hydroxymethylcytosine (5hmC), 5-formylcytosine (5fC), and 5-carboxylcytosine (5caC).^6^ The formation of 5fC and 5caC allows for their active removal by thymine DNA glycosylase (TDG) through the base excision repair (BER) pathway, facilitating active DNA demethylation.^7^ Recently, 5hmC has gained significant attention, being recognized as the sixth base in the mammalian genome.^8^ As the second most abundant DNA modification following 5mC, 5hmC is now acknowledged to be a distinct epigenetic mark with functions that contrast with those of 5mC.^9–11^

Several base-conversion chemistries are used to differentiate unmodified cytosine (C) from its epigenetic variants. These methods include: (1) bisulfite-based methods like whole-genome bisulfite sequencing (WGBS), where bisulfite treatment converts C to U, but not 5mC or 5hmC;^12^ and (2) bisulfite-free techniques such as enzymatic methyl-sequencing (EM-seq), using Tet2 to oxidize 5mC and 5hmC to 5caC, followed by A3A treatment, which converts C to U, excluding the newly generated 5caC.^13^ This method can be optimized for single-cell 5mC detection.^14^ In TET-assisted pyridine borane sequencing (TAPS), Tet2 oxidizes 5mC and 5hmC to 5caC, and then borane reduction converts 5caC to DHU.^15^ However, traditional methods for measuring 5mC often fail to distinguish between 5mC and 5hmC. Thus, several base-conversion techniques have been developed to separate 5mC and 5hmC, such as oxidative bisulfite sequencing (oxBS-seq) and Tet-assisted bisulfite sequencing (TAB-seq) under bisulfite-based methods,^16,17^ as well as APOBEC-coupled epigenetic sequencing (ACE-seq),^18^ chemical-assisted C-to-T conversion of 5hmC sequencing (hmC-CATCH),^19^ chemical-assisted pyridine borane sequencing (CAPS) in bisulfite-free approaches,^20^ and single-step deamination sequencing (SSD-seq).^21^ OxBS-seq employs potassium perruthenate for the oxidation of 5hmC to 5fC, followed by bisulfite treatment.^16^ TAB-seq uses beta-glucosyltransferase (β-GT) to safeguard 5hmC, while Tet2 oxidizes 5mC to 5caC.^17^ ACE-seq also utilizes β-GT for 5hmC protection, then applies A3A treatment to deaminate C and 5mC.^18^ hmC-CATCH involves potassium ruthenate oxidation of 5hmC to 5fC, followed by indanedione labeling.^19^ Similarly, CAPS uses potassium ruthenate for 5hmC oxidation to 5fC and then employs borane reduction to convert it to DHU.^20^ SSD-seq utilized a screened engineered A3A protein (eA3A-v10) to selectively deaminate C and 5mC, but not 5hmC.^21^ Each method provides unique approaches for accurate differentiation of these epigenetic marks. These approaches are crucial in detecting and differentiating various DNA modifications, enhancing our understanding of epigenetic regulation.

5mC and 5hmC are different types of DNA modifications that play important roles in epigenetic regulation. 5mC is concentrated in promoter regions and is associated with repressive gene expression.^2,22^ However, 5hmC, concentrated in specific genomic regions, plays a unique role in gene expression and cellular functions.^23,24^ An example of its importance is observed with the methyl-CpG binding protein 2 (MECP2), where mutations cause Rett syndrome.^25^ MECP2 binds strongly to 5mC but not to 5hmC in the CG context, impacting epigenetic regulation through its differential affinity.^26,27^ Therefore, distinguishing between 5mC and 5hmC is crucial for accurately measuring cell-type-specific epigenomic profiles in health and disease contexts. Thus, this review aims to summarize methods for simultaneously profiling 5mC and 5hmC based on next-generation sequencing methods and to discuss the challenges, limitations, and potential prospects.

## 2. Detection methods based on restriction-enzymes

These methods utilize various types of restriction enzymes, capitalizing on their different responses to specific DNA modifications. This approach enables selective detection by leveraging the unique activities of these enzymes towards distinct epigenetic marks.

### DARESOME

The DARESOME method achieves simultaneous profiling of 5mC and 5hmC by using a sequence of enzymatic reactions and DNA tagging.^28^ This process involves three key steps: (1) initial digestion and tagging: the DNA is first digested using the Hpa II enzyme, which targets unmodified CCGG sites, and then tagged with specific adapters (U-tags). (2) Secondary digestion and tagging: the remaining CCGG sites, which may contain 5mC, or 5hmC, are digested with Msp I enzyme and tagged with H-tags. (3) Glycosylation and final tagging: 5hmC bases are glycosylated to form β-glucosyl-5-hydroxymethyl-cytosine (5gmC), protecting them from further digestion. A second Msp I digestion is then performed, followed by tagging with M-tags for fragments containing 5mC. These steps result in tagged DNA fragments, which can be sequenced to identify the modification states of CCGG sites across the genome. The use of different tags for unmodified cytosine, 5mC, and 5hmC allows for their simultaneous detection and differentiation. However, this method primarily captures CCGG sites, representing only about 10% of all CG sites, thus limiting its scope. Furthermore, the reliance on restriction-enzyme cutting restricts the analysis to only one site per DNA fragment, making it challenging to analyze multiple neighboring 5mC and 5hmC sites within short ranges. This highlights a significant limitation in the method's ability to provide comprehensive epigenetic profiling.

### Dyad-seq

**Fig. 1:**
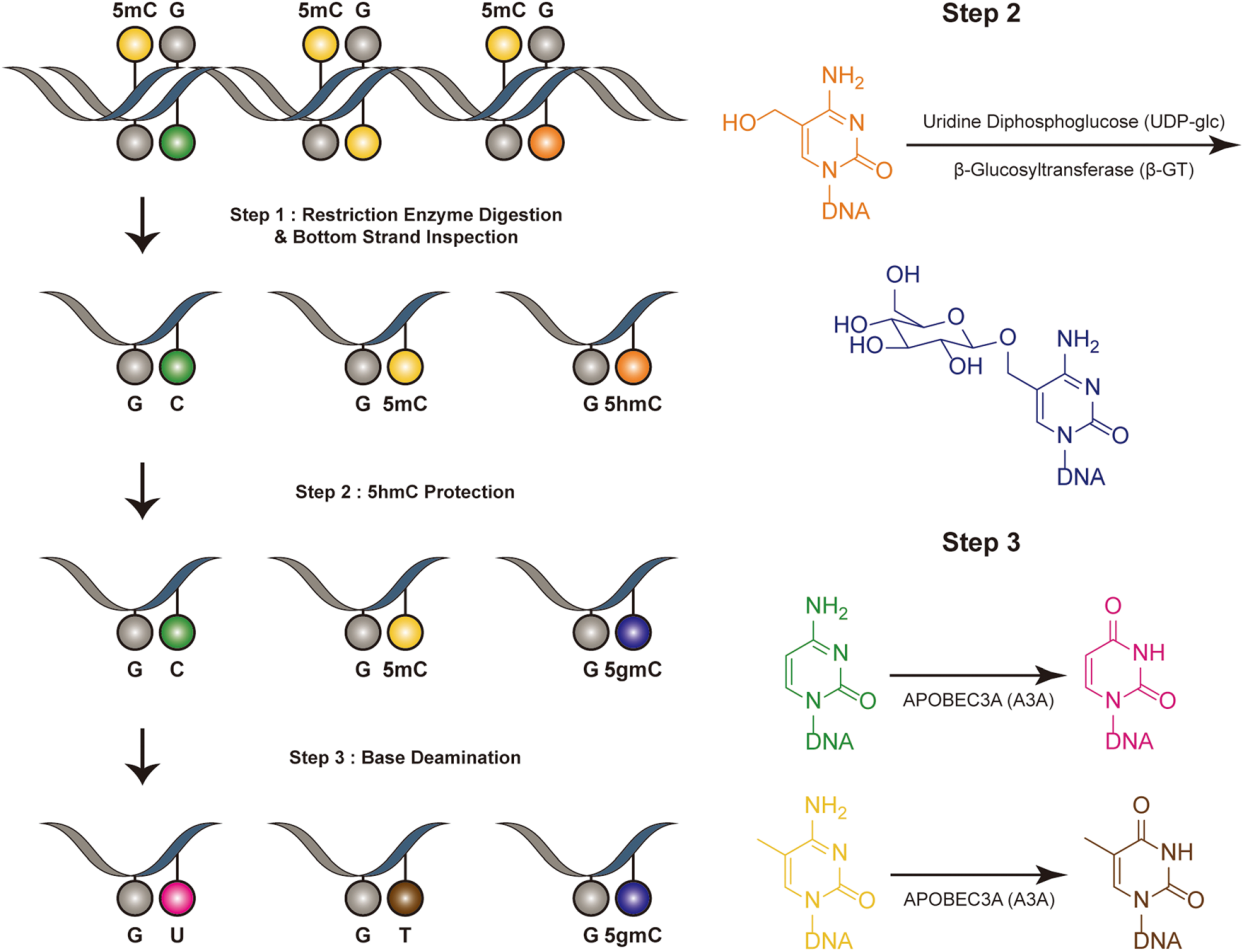
Overview of dyad-seq. This figure illustrates the schematic and sequencing features of the dyad-seq for combined detection of 5mC and 5hmC.

Dyad-seq combines enzymatic detection of modified cytosines with traditional nucleobase conversion techniques^29^ (Fig. 1). It is designed to quantify all combinations of 5mC and 5hmC at individual CpG dyads. Dyad-seq utilizes the different methods to detect the DNA modifications on different strand. Firstly, it uses two restriction enzymes, MspJI and AbaSI, to digest DNA. MspJI is a unique restriction endonuclease recognized 5mC on the top strand.^30^ It distinctively cleaves DNA at a fixed distance from a 5mC site. AbaSI is a specialized modification-dependent restriction endonuclease that targets DNA containing 5hmC and 5gmC on the top strand.^31^ Following the digestion of DNA with MspJI or AbaSI, the bottom strand of the fragmented DNA molecules is captured through ligation to a well-designed adapter, which is specific liagted to the bottom strand. This adapter contains a random overhang, a sample barcode, a unique molecule identifier (UMI), and a sequence for PCR amplification. Next, to identify methylated cytosines on the opposing DNA strand, the samples are treated in one of two ways. They are either enzymatically processed with Tet2, β-GT and A3A or they undergo sodium bisulfite treatment, which specifically converts unmodified cytosines into uracil, while methylated cytosines remain read as C (M-M-dyad-seq and H-M-dyad-seq). Moreover, a minor adjustment to the enzymatic conversion reaction, involving β-GT and A3A, specifically allows for the detection of 5hmC on the opposite DNA strand (M-H-dyad-seq and H-H-dyad-seq). Each combination uniquely targets and identifies specific cytosine modifications, facilitating comprehensive analysis. The M-M-dyad-seq method was adopted for single-cell methylome detection and further integrated with single-cell RNA-seq. This method, termed scDyad &T-seq, enables the simultaneous analysis of both the transcriptome and the methylome at the single-cell level. This method offers multiple assays for simultaneously detecting 5mC or 5hmC, and it can simultaneously quantify genome-wide methylation levels and mRNA from the same cells. However, this method, like DARESOME, depends on restriction-enzyme cutting, which limits its ability to detect only one site per DNA fragment. This restriction prevents the analysis of multiple neighboring 5mC and 5hmC sites in close proximity, representing a significant limitation in understanding the complex patterns of these epigenetic marks. Additionally, the presence of natural DNA breaks in the sample can lead to false positive results, presenting a challenge for accurate interpretation of the data.

## 3. Detection methods based on chemical processing

Innovative chemical reactions play a crucial role in developing new epigenome sequencing tools. This section highlights chemical methods for simultaneously detecting 5mC and 5hmC, emphasizing the importance and potential of these techniques in advancing our understanding of the epigenome and its implications in various biological processes and diseases.

### Six-letter-seq

**Fig. 2:**
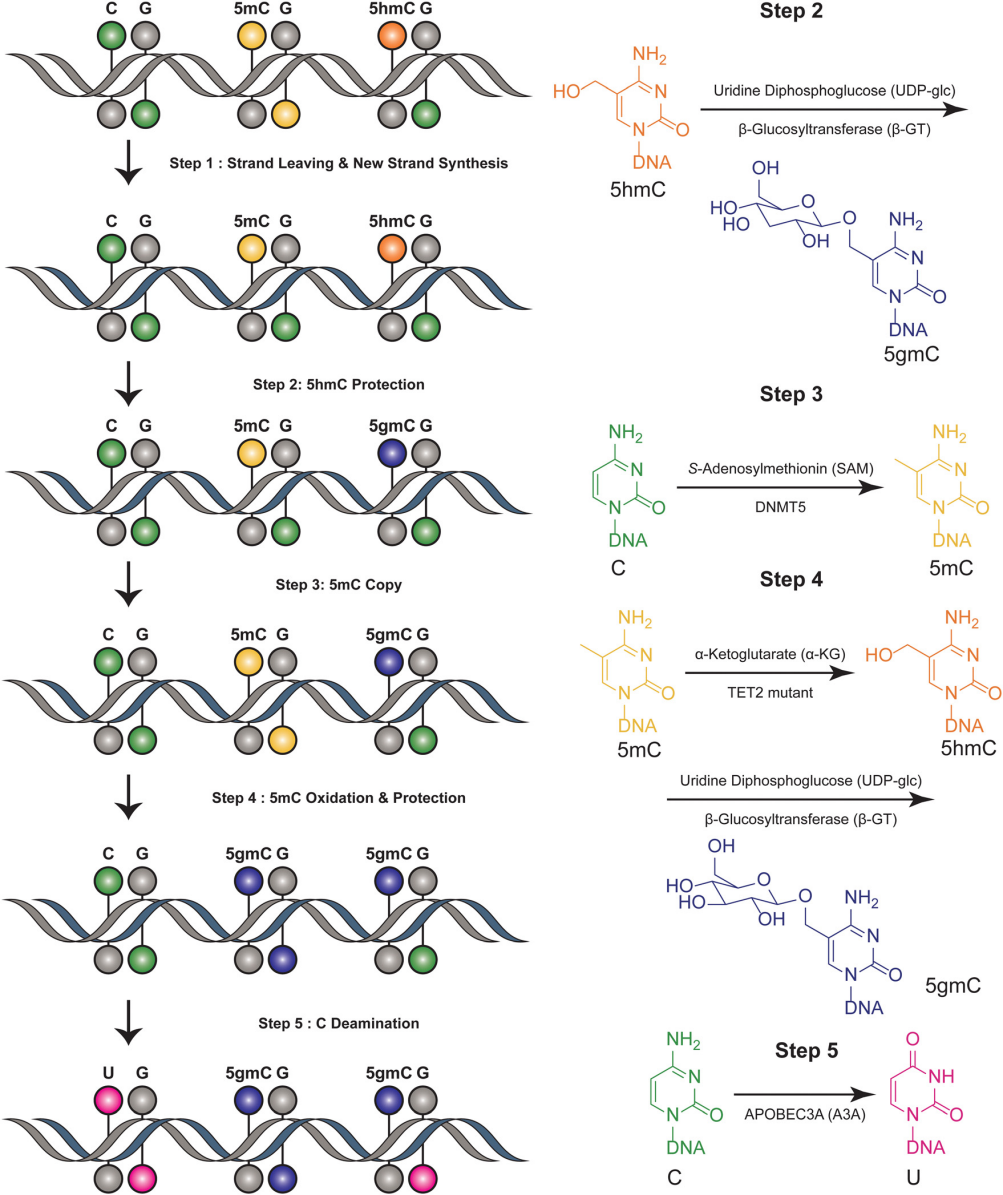
Overview of six-letter-seq. This figure illustrates the joint profiling of genetics and epigenetics information.

The six-letter seq workflow, designed to resolve the four genetic bases and the epigenetic modifications 5mC and 5hmC, involves a series of sophisticated steps^32^ (Fig. 2). It starts with the fragmentation of DNA, followed by ligation with synthetic DNA hairpin adapters. The strands are separated, and a complimentary copy strand, lacking epigenetic modifications, is synthesized by a synthetic hairpin. A pivotal aspect of the six-letter seq method is its ability to accurately distinguish between 5mC and 5hmC, while simultaneously ensuring precise genetic base calling within the same DNA fragment. DNA methylation modifications are added to the CpG position of newly synthesized DNA strands that do not contain modification information, utilizing DNA methyltransferase 5 (DNMT5) for its specificity in copy methylation.^33^ Concurrently, 5hmC is safeguarded from this copying process through glycosylation, executed by beta-glycosyltransferase.^34^ This selective glycosylation effectively prevents the replication of 5hmC modifications onto the copy strand, enabling a distinct differentiation between 5mC and 5hmC in the sequencing analysis. Additionally, the 5mC is enzymatically oxidized by a TET2 mutant^35^ and subsequently uses β-GT to protect all 5hmCs. Furthermore, the cytosines are deaminated to uracil by APOBEC3A(A3A).^36^ Meanwhile, UvrD helicase is added to facilitate the generation of a single-stranded DNA substrate, which is necessary for the efficient action of A3A.^37^ The DNA constructs are sequenced using a paired-end format. This involves read 1, primed by P7, representing the original DNA strand, and read 2, primed by P5, representing the synthesized copy strand. These reads are then aligned pairwise, ensuring that read 1 is matched with its complementary read 2. This alignment is critical for accurately reconstructing the sequence and identifying both genetic and epigenetic information from the DNA sample. Computational alignment generates a resolved read, which is then aligned to the reference genome for genetic variant and methylation analysis. This method ensures accurate differentiation between genetic and epigenetic information in DNA analysis. This method can distinguish between four bases and their epigenetic information, using the complementary DNA strand for correction. However, it is not suitable for single-cell detection due to its complex procedural steps. This limitation highlights a trade-off between the method's comprehensive detection capabilities and its practical applicability in more streamlined or single-cell contexts.

### Joint-snhmC-seq

**Fig. 3:**
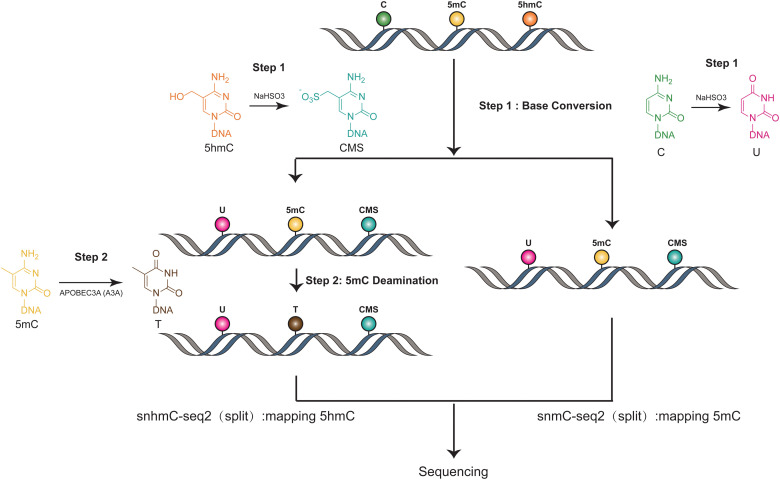
Overview of Joint-snhmC-seq. The figure demonstrates the concurrent profiling of 5mC and 5hmC at the single-cell level.

The methods used in Joint-snhmC-seq involve a combination of technologies and processes to simultaneously profile 5mC and 5hmC in single cells^38^ (Fig. 3). First, lysed individual cells/nuclei were treated with bisulfite, which can potentially achieve chemical protection of 5hmC through the formation of cytosine-5-methylenesulfonate (CMS) and can chemically modify cytosine to uracil.^39^ The converted single-stranded DNA (ssDNA) is divided into two portions. One part is used directly to detect signals of 5mC and 5hmC(snmC-seq2). The other part undergoes an optimized enzymatic deamination process, typically using A3A. This enzymatic treatment is efficient in converting 5mC to thymine (T), facilitating the differentiation and analysis of these epigenetic marks. After enzymatic deamination using A3A, the treated single-stranded DNA (ssDNA) can be effectively captured through random priming or post-deamination adapter tagging(snhmC-seq2). This step is crucial for enabling low-input bulk or single-cell 5-hydroxymethylome sequencing analysis, enhancing the method's efficiency and applicability for detailed epigenetic studies. The analysis of APOBEC3A-deaminated ssDNA is concurrently conducted by two separate methods: snhmC-seq2 for mapping 5hmC and snmC-seq2 for identifying true 5mC, achieved by subtracting 5hmC signals from the combined 5hmC and 5mC signals. This dual approach, by physically linking two distinct epigenetic modalities—5hmC and true 5mC—from the same cell, effectively bypasses the challenge of cross-modal computational integration. This is a significant advancement for reconstructing single-cell 5hmC and 5mC profiles. However, this method's indirect approach in detecting 5mC and 5hmC can lead to false positives if enzyme efficiency is not optimal. Additionally, the conversion of all cytosines to uracil reduces sequence complexity, which may result in a lower mapping ratio. Moreover, this technique necessitates dividing genomic materials into two separate parts for analysis, adding complexity to the process. These factors underscore the need for careful consideration of methodological limitations in epigenetic studies.

### SIMPLE-seq

**Fig. 4:**
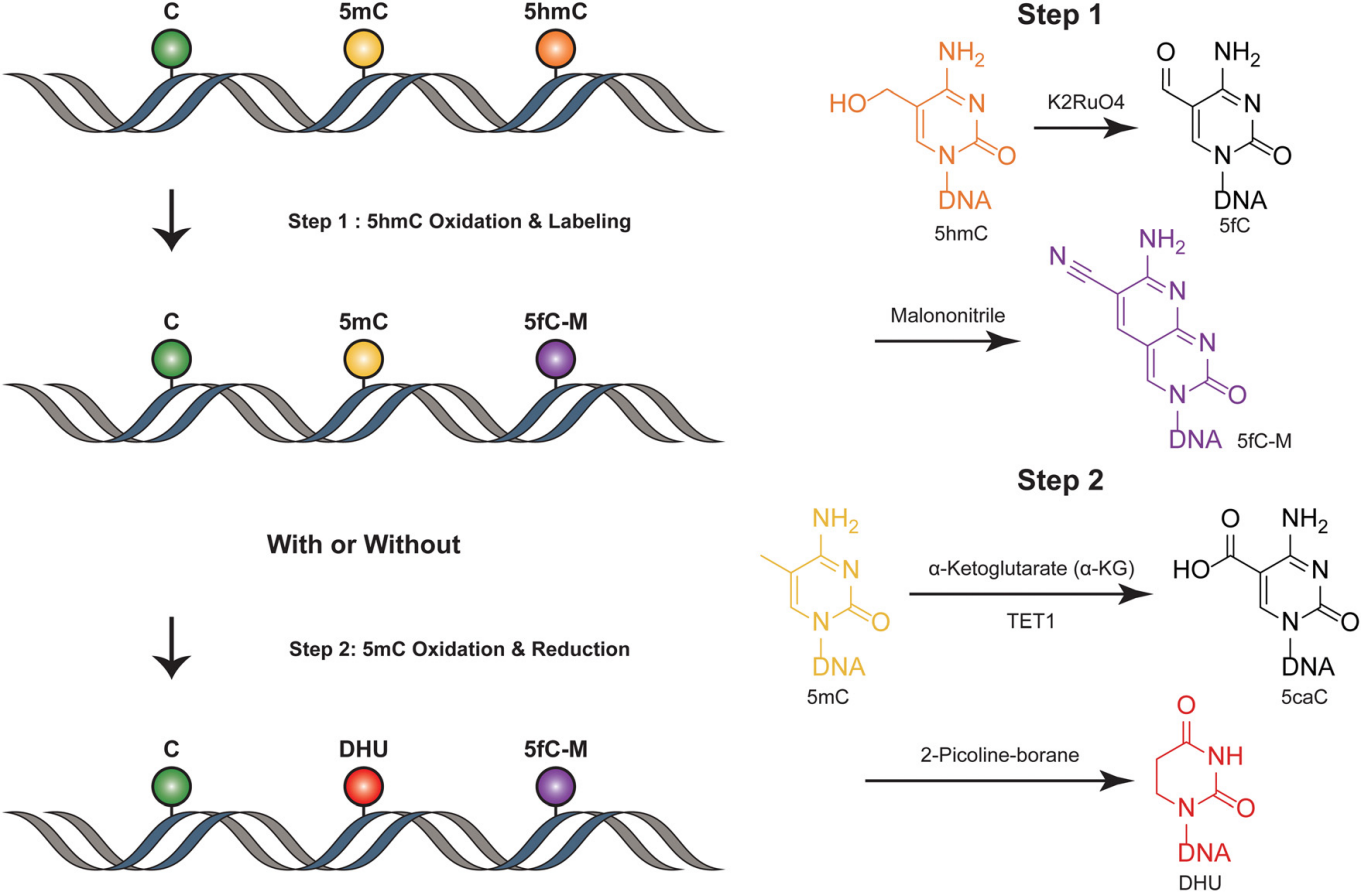
Overview of SIMPLE-seq. The figure depicts the concurrent profiling of 5mC and 5hmC within a single molecule.

SIMPLE-seq is a high-throughput method for the simultaneous detection of 5mC and 5hmC in single cells.^40^ In this process, ruthenate (VI) oxidizes 5hmC to 5fC, which is then labeled by malononitrile^19,41^ (Fig. 4). This generates a specific “20C-to-T” signal at 5hmC sites after PCR amplification, while leaving C and 5mC sites unchanged. Primer extension is used to record this “5hmC-to-T” transition on the complementary strand. The subsequent step involves TET-mediated oxidation, which transforms 5mC in the original DNA template into 5caC. This 5caC is then subjected to borane reduction, resulting in the formation of DHU.^15^ This process is crucial as it generates a second “C-to-T” signal specifically at the 5mC sites within the same DNA molecule, facilitating the differentiation and identification of these sites. Unmodified cytosines and other bases remain unaffected throughout this process. To differentiate between 5mC and 5hmC, both of which produce “C-to-T” mutations, a specific primer pre-deposited with a 5caC base is designed to record the 5mC signals in the extension products. During the subsequent reaction targeting 5mC, this 5caC base is converted to a “T” signal. This transformation is key in distinguishing between the amplification products derived from 5mC and 5hmC, allowing for accurate identification and analysis of these epigenetic modifications in the DNA template. SIMPLE-seq is scalable and offers base-resolution analysis of both modifications. This allows the identification of the types and locations of modifications from the same DNA molecule in single cells. However, in scenarios where endogenous 5fC is labeled and undergoes a C-to-T transition, it is incorrectly identified as 5hmC, leading to potential false positive results.

## 4. Discussion

Various studies suggest that increased DNA methylation in promoter regions typically correlates negatively with gene expression.^42,43^ However, single-cell multi-omics sequencing indicates that this negative association between promoter methylation levels and gene expression is only evident in a small percentage of cases.^44–46^ A potential reason for this discrepancy could be the inability of previous detection methods to differentiate between 5mC and 5hmC. This limitation might lead to mixed signals, thereby obscuring the true relationship between 5mC levels and gene expression. Thus, distinguishing between 5hmC and true 5mC enhances our understanding of gene regulation. Furthermore, the dynamic changes of 5mC and 5hmC during cell fate transition can well explain how these two modifications vary in the process of cell fate change. The dual-modality epigenetic sequencing approach provides clarity in distinguishing between 5mC and 5hmC, addressing the challenges posed by traditional DNA methylome sequencing methods that often conflate these two modifications. This enhanced resolution in identifying true 5mC and 5hmC profiles significantly improves the accuracy of integrating multimodal data, thereby offering a more precise understanding of epigenetic modifications and their implications. This advancement marks a significant step forward in the field of epigenetics.

Recently, the emergence of spatial omics has significantly enhanced our understanding of disease mechanisms through the spatial mapping of cells within tissues.^47^ Nonetheless, the scope of current spatial technologies predominantly focuses on gene expression levels, leaving other aspects like the epigenome less explored. This highlights the necessity for further advancements in spatial omics, aiming to incorporate a broader range of omics data such as epigenomic information, for a more comprehensive and accurate profiling of tissue structures.

Moreover, these methods focusing on 5mC and 5hmC could be integrated with single-molecule multiplexed detection methods for different modifications. With further modification, it has the potential for extensive single-cell multimodal integration, including transcriptome, 3D genome structure, chromatin states, and protein abundances.^48–59^ This approach opens up possibilities for comprehensive analysis and understanding of cellular processes at the single-cell level by monitoring various molecular layers simultaneously. Progress in the field of multi-omics will significantly contribute to the development of sophisticated therapeutic strategies. Additionally, it will enable the creation of comprehensive atlases that encompass various omics layers and temporal scales, enhancing our understanding of health and disease.

Furthermore, in recent years, an increasing number of studies have indicated that 5mC and 5hmC can serve as biomarkers for early cancer screening.^60–67^ 5mC exhibits significant up-regulation, particularly at certain key oncogenes, while 5hmC experiences a notable decrease in tumors.^66^ Accurately distinguishing the signal changes of 5mC and 5hmC can significantly improve the precision of tumor diagnosis. These methods provide a precise and effective technique for future disease detection.

## Conflicts of interest

The authors declare that they have no conflicts of interest in this work.

## Supplementary Material
